# SG-APSIC1085: Microbiological analysis concerning antimicrobial effect of atomized ionless hypochlorous acid water in a hospital environment

**DOI:** 10.1017/ash.2023.39

**Published:** 2023-03-16

**Authors:** Miho Miura, Hideki Katayama, Atsushi Miyake, Toru Sakamoto, Tetsuya Naitou, Yoshiro Sakai, Chiyoko Tanamachi, Kenji Goto, Hiroshi Watanabe

**Affiliations:** Kurume University Hospital, Kurume, Japan; Kurume University Hospital, Kurume, Japan; Kurume University Hospital, Kurume, Japan; Kurume University Hospital, Kurume, Japan; Kurume University Hospital, Kurume, Japan; Kurume University Hospital, Kurume, Japan; Kurume University Hospital, Kurume, Japan; Kurume University Hospital, Kurume, Japan; Kurume University Hospital, Kurume, Japan

## Abstract

**Objectives:** We evaluated the disinfecting efficacy of atomized ionless hypochlorous acid water (CLFine) against pathogenic microorganisms in an isolation room. **Methods:** The study was conducted in an isolation room of Kurume University Hospital. CLFine with available chlorine concentrations of 40 ppm and 300 ppm as test substances and purified water as control were atomized with an ultrasonic atomizer (CLmistL). The 40 ppm and 300 ppm of CLFine were atomized at the atmospheric available chlorine concentrations of ~0.03 ppm and 0.1~0.2 ppm, respectively, and purified water was atomized in the same manner as CLFine. Petri dishes with *Staphylococcus aureus, Bacillus cereus* spores, *Bacillus subtilis* spores and *Aspergillus ruber* were allocated in the room, then CLFine or purified water was atomized. Sampling was performed at 3 and 5 hours after the start of atomization, and the bacterial counts were measured. The study was carried out either with air conditioning turned “on” or “off” because atmospherically available chlorine concentration is affected by ventilation. **Results:** When the air conditioning was turned on, purified water showed a slight reduction of bacterial counts by 0.9 log or less at 5 hours after the atomization. When CLFine was used, 40 ppm greatly reduced the counts of *Staphylococcus aureus* by 5.1~5.4 logs reduction at 5 hours after the atomization, but no distinctive efficacy was observed against other microorganisms. On the other hand, 300 ppm caused a significant reduction of the bacterial counts for all the microorganisms at 5 hours after the atomization (*P* < .001 vs purified water). The same results were observed in the environment with the air conditioning turned off. **Conclusions:** Our data suggest that CLFine effectively disinfects pathogenic microorganisms and can contribute to maintaining the hygienic environment of hospital rooms. This study was funded as contracted research by NIPRO Corporation with the approval of the ethics committee (study no. 21229).

